# Efficacy of different load intensity and time-under-tension calf loading protocols for Achilles tendinopathy (the LOADIT trial): protocol for a randomised pilot study

**DOI:** 10.1186/s40814-020-00639-5

**Published:** 2020-07-13

**Authors:** Fatmah Hasani, Terry P. Haines, Shannon E. Munteanu, Bill Vicenzino, Peter Malliaras

**Affiliations:** 1 Physiotherapy Department, School of Primary and Allied Health Care, Faculty of Medicine Nursing and Health Sciences, Monash University, Frankston, Victoria 3199 Australia; 2 Physiotherapy Department, Security Forces Hospital Program, Riyadh, 11481 Kingdom of Saudi Arabia; 3 School of Primary and Allied Health Care, Faculty of Medicine Nursing and Health Sciences, Monash University, Frankston, Victoria 3199 Australia; 4 Discipline of Podiatry, School of Allied Health, Human Services and Sport, College of Science, Health and Engineering, La Trobe University, Melbourne, Victoria 3086 Australia; 5 La Trobe Sport and Exercise Medicine Research Centre, School of Allied Health, Human Services and Sport, College of Science, Health and Engineering, La Trobe University, Melbourne, Victoria 3086 Australia; 6 Sports Injuries Rehabilitation and Prevention for Health Research Unit, School of Health and Rehabilitation Sciences, The University of Queensland, Brisbane, Queensland Australia

**Keywords:** Achilles tendinopathy, Rehabilitation, Load intensity, Time-under-tension, Telerehabilitation

## Abstract

**Background:**

Modifying variables in exercise prescription can produce specific effects on Achilles tendinopathy outcomes. This study aims to determine the feasibility of conducting an adequately powered randomised trial in the future to assess the efficacy of different load intensity and time-under-tension exercise parameters for improving pain and function in individuals with persistent midportion Achilles tendinopathy.

**Methods:**

The trial is designed as prospective, four-armed feasibility and randomised pilot trial with 3 months follow-up. Interventions will be provided in a gym setting. The investigator, who will be blind to the allocation of participants, will conduct all pre- and post-intervention assessments. Forty-eight male participants with Achilles tendinopathy will be recruited from the community. We will use a 2 × 2 factorial design with factors of load intensity (six or eighteen repetitions maximum) and time-under-tension (two or six second repetitions). Participants will be randomised into one of the testing groups: six RM with two second repetitions, six RM with six second repetitions, eighteen RM with two second repetitions or eighteen RM with six second repetitions. Trial feasibility will be indicated by the rate of conversion, recruitment and retention, adherence to the interventions by participants, the utility of videoconferencing mode for weekly exercise supervision, incidence of adverse events, and feasibility of future economic evaluation. The secondary clinical outcomes will assess pain and disability, participant impression of change, satisfaction, health-related quality of life, physical activity, work absenteeism, psychological measures at baseline, 6 and 12 weeks, and plantarflexor contractile dysfunction (torque, rate of force development and muscle force steadiness) at baseline and 12 weeks. These clinical outcomes are primarily measured to provide information regarding potential treatment effects and trends.

**Discussion:**

The proposed study and follow-up powered randomised trial will be a first step towards determining exercise dose parameters that may optimise outcomes for Achilles tendinopathy. We have chosen to focus on load intensity and time-under-tension, as these parameters are important for tendon adaptation. This work has the potential to lead to more effective exercise loading interventions for Achilles tendinopathy.

**Trial registration:**

Australian New Zealand Clinical Trials Registry, ACTRN12618001315202. Registered retrospectively on August 6th, 2018.

## Background

Achilles tendinopathy is an overuse injury characterised by localised Achilles tendon load-related pain and dysfunction. This condition is often associated with a reduced ability to walk and run, which has consequential effects on general health and wellbeing [1, 2]. Achilles tendinopathy is estimated to account for up to 18% of all running injuries [3] and has an estimated cumulative lifetime prevalence of 6% in the general population [4]. The aetiology of tendinopathy is multifactorial [5], one major factor being the imbalance between load demands placed on the tendon and its ability to remodel [6]. Other factors influencing the capacity of the tendon to remodel, and thereby increase the risk of tendinopathy, include older age, genetic profile, and metabolic factors such as elevated cholesterol or diabetes. The pain mechanisms in tendinopathy are unknown and many potential structural (e.g. neurovascular ingrowth) and biochemical (e.g. changes in metabolites or cytokines) mechanisms may contribute [7].

Clinical practice guidelines, informed by systematic reviews [8, 9], recommend calf muscle exercise as a first-line treatment for Achilles tendinopathy [10]. Eccentric calf muscle loading, as originally described by Alfredson, has been—and continues to be—a popular conservative intervention for Achilles tendinopathy [11]. The intervention involves progressive, heavy isotonic exercise; only the eccentric phase is performed. Other popular isotonic resistance loading programs include the Silbernagel combined and heavy slow resistance programs [12, 13]. The Silbernagel combined program [14] involves progression from eccentric-concentric to eccentric loading, and finally to faster or plyometric type eccentric-concentric loading. Exercise is performed once daily, rather than twice as with Alfredson eccentric loading. The heavy slow resistance program [13] involves high load, progressive gym-based loading for the calf muscles performed three times per week. Comparing the efficacy of these pragmatic protocols, there is currently insufficient evidence to conclude that there is an optimal exercise protocol for Achilles tendinopathy [8, 13, 15].

The difficulty in comparing existing exercise programs for Achilles tendinopathy is that several exercise parameters vary—such as volume (number of repetitions or sets performed), load intensity (e.g. % one repetition maximum), and frequency (number of exercises performed per day)—or they are incompletely reported. Consequently, determining the dose parameters that confer the greatest benefit is not possible. For example, Beyer et al. [13] compare eccentric training and heavy slow resistance, though the latter involved lower volume and higher load intensity. Therefore, there is a need to evaluate the efficacy of calf loading exercise with varying dose parameters in people with Achilles tendinopathy.

Several variables can be modified in exercise prescription to produce specific effects on the musculotendinous system and, potentially, Achilles tendinopathy outcomes. Some modifiable variables include load intensity, time-under-tension, speed of contraction, rest between sets and frequency of exercise sessions and position of the limb. It is well established that the tendon is a mechanoresponsive tissue, and that the key driver for tendon adaptation appears to be tissue strain [16]. Given tendon tissue strain is proportional to tendon load and the amount of time this load is applied within a specific contraction (time-under-tension), it follows that these parameters will determine strain and, hence, tendon adaptation [16]. A recent systematic review of loading protocols that result in tendon adaption concluded that load intensity is the key determinant of tendon tissue adaptation to load; the type of contraction—for example, eccentric versus concentric—did not influence adaptation [17]. Although time-under-tension and load intensity are known to have specific effects on tendon adaptation, the influence of these parameters on clinical outcomes in Achilles tendinopathy is unknown. This makes examining the efficacy of load intensity and time-under-tension on Achilles tendinopathy outcomes a logical first step.

### Aims

This study aims to assess the feasibility of a future definitive randomised trial to examine the efficacy of different LOAD-intensity and time-under-tension (LOADIT) exercise parameters for Achilles tendinopathy. The primary objective is to assess the feasibility of study processes, including (i) rate of participant recruitment, conversion, and retention; (ii) ability to perform the interventions per protocol (adequate exercise fidelity and adherence based on weekly assessment via videoconferencing mode); (iii) incidence and type of adverse events; (iv) feasibility of future economic evaluation; and (v) acceptability of telerehabilitation videoconference sessions. The secondary objective is to explore trends in treatment effects and variability—in pain and disability, participant perception of change, satisfaction, health-related quality of life, physical activity, work absenteeism, psychological measures, and plantar flexion contractile dysfunction—between the groups at 6 and 12 weeks. These time points have been selected based on evidence that exercise effect should be maximised at 12 weeks [18].

## Methods

### Trial design

**Fig. 1:**
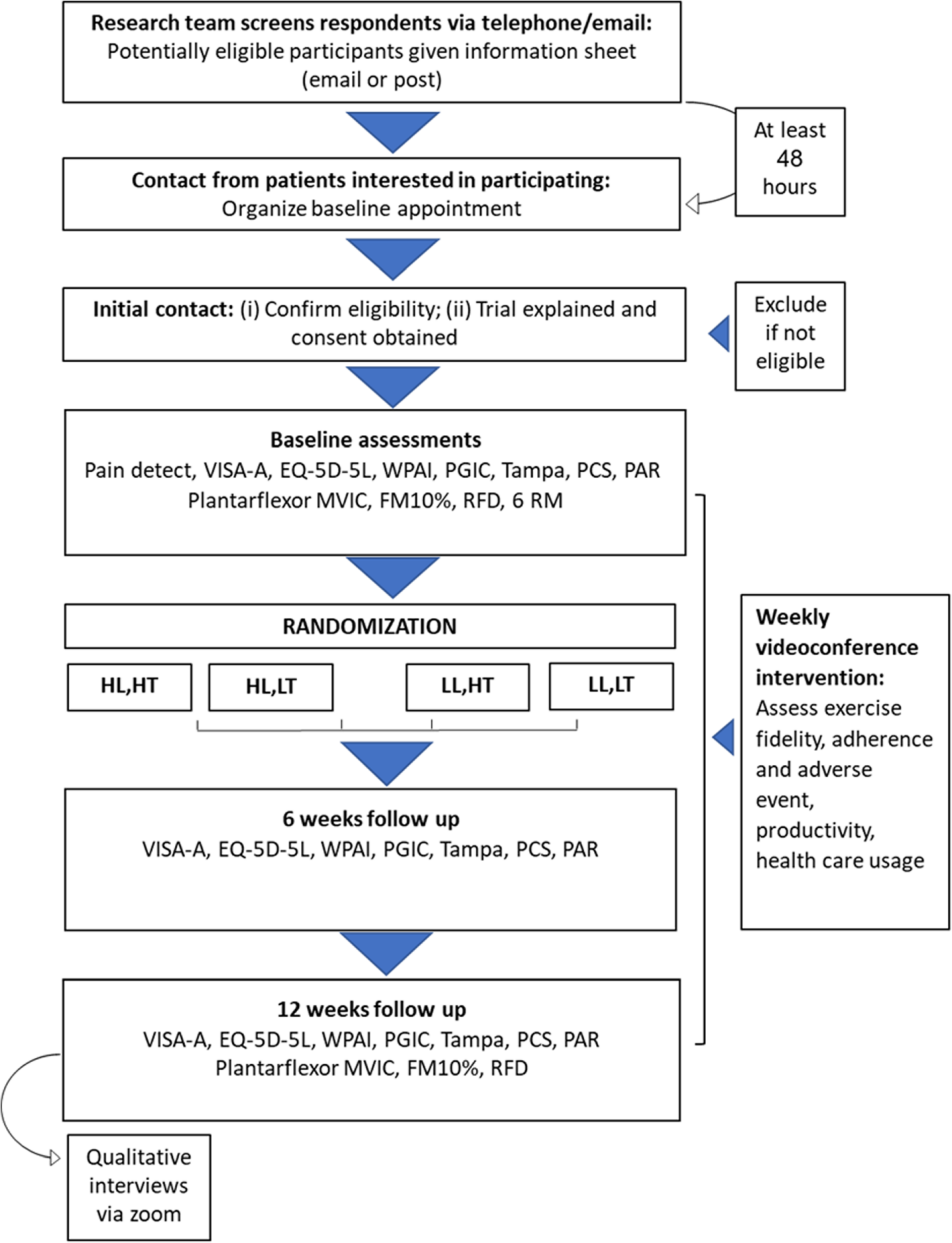
Trial profile. HL: High Load; HT: High time-under-tension; LL: Low load; LT: Low time-under-tension; VISA-A: Victorian Institute of Sports Assessment–Achilles; EQ-5D-5L: Health-related quality of life; WPAI: Work Productivity and Activity Impairment; PGIC: Patient Global Impression of Change; PCS: Pain Catastrophising Scale; MVIC: Maximal Voluntary Isometric Contraction; FM10%: Force Match at 10%; RFD: Rate of Force Development; RM: Repetition Maximum

LOADIT is a four-arm, factorial randomised pilot study with a nested qualitative study. The factorial design is a 2 × 2: the two factors are load intensity and time-under-tension, and each has two levels (higher versus lower). Outcome assessment will occur at baseline, 6 and 12 weeks (Fig. 1). The study protocol has been reported using the Standard Protocol Items: Recommendations for Interventions (SPIRIT) statement guidelines [19] and the publication associated with the trial’s findings will be reported in accordance with the CONSORT extension for randomised pilot and feasibility trials. The protocol has been registered at the Australian New Zealand Clinical Trials Registry, ACTRN 12618001315202.

### Participant recruitment and eligibility criteria

Participants will be recruited via social media (i.e. Facebook, Twitter), and by posting study information on appropriate websites (e.g. running clubs and forums), at local community centres, senior citizen centres and sports clubs. Health professionals in Melbourne that manage Achilles tendinopathy will be asked to refer potentially eligible participants. The recruitment window is anticipated to be 6 to 8 months.

Respondents will be initially screened via telephone or email by researchers. Potentially eligible participants that satisfy the selection criteria will be provided with study information electronically and given at least 48 h to decide whether they would like to proceed.

### Inclusion criteria

Participants must meet the following criteria:

(i) Male aged 18 to 70 years.
(ii) Have a history of Achilles tendon pain for 12 weeks or more.
(iii) Current midportion Achilles tendinopathy in one or both lower limbs, as determined by the satisfaction of all of the following criteria (based on current recommendations [20]); (a) insidious onset of pain 2 to 6 cm above the Achilles tendon insertion; (b) pain with or after weight-bearing activities, which is worse in the morning or upon weight-bearing after a period of rest; (c) ultrasound imaging consistent with Achilles tendinopathy (anteroposterior thickness and/or hypoechoic regions within the midportion of the Achilles tendon).
(iv) A score of ≤75 on the Victorian Institute of Sports Assessment—Achilles questionnaire (VISA-A).
(v) Willing to attempt to access a gym three times per week to perform the exercise interventions.
(vi) Willing to abstain from treatments other than the study interventions for Achilles tendon pain during the study period.
(vii) Competent in both written and spoken English, and able to provide informed written consent.

### Exclusion criteria

Potential participants will be excluded if any of the following criteria are present:

(i) Previous Achilles tendon rupture or surgery in symptomatic lower limb(s).
(ii) Other diagnosis of Achilles tendon pain that is not midportion Achilles tendinopathy, such as impingement syndrome, insertional Achilles tendinosis, or Achilles paratenonitis.
(iii) Inflammatory arthritis, such as rheumatoid arthritis or ankylosing spondylitis.
(iv) Metabolic and endocrine disorders, such as type I or II diabetes.
(v) Neurological disorders affecting the lower limb, such as multiple sclerosis.
(vi) Use of fluoroquinolone antibiotics within the previous 2 years.
(vii) Current or recent (i.e. within the last 3 months) strength exercise treatment for Achilles tendon pain.
(viii) Injection of pharmaceutical agent for Achilles tendon pain in the last 3 months.
(ix) Injury of lower limb(s) or back that may interfere with the execution of exercise interventions in the study, such as fractures.

### Randomisation

Participants will be randomised to receive one of four exercise interventions. The first two arms involve lower load intensity, where participants will perform four sets of eighteen repetitions per set prior to failure (i.e. inability to do another full repetition) with either high or low time-under-tension. The second two arms involve higher load intensity, where participants will perform four sets of six repetitions per set prior to failure with high or low time-under-tension. Time-under-tension in this context refers to how long the calf muscle-tendon unit is under load. This will be exactly 6 s (3 s concentric phase, 3 s eccentric phase) in the high time-under-tension group and 2 s (1 s concentric phase, 1 s eccentric phase) in the low.

Permuted blocks of variable and undisclosed block sizes (four, eight and twelve) will be used to randomise participants to a treatment group. The allocation sequence will be computer generated and entered into sealed opaque envelopes by a researcher at a remote location who is not directly involved with trial recruitment.

### Blinding

Due to the nature of the interventions, participants and clinicians providing care will be aware of treatment allocation. Participants will be told that researchers are testing four different exercise protocols and that there is uncertainty about which is more effective. Administered and collected outcome measures will be done by a single investigator, who will be blind to treatment allocation. Research staff entering and analysing data collected in a coded data file will also be blind to allocation and uninvolved in any interventions.

### Screening

Those wishing to participate will be scheduled to attend a baseline assessment at Monash University in Melbourne, Australia, where the investigator (FH)—a qualified physiotherapist trained in performing Achilles tendon ultrasound imaging—will assess eligibility, perform baseline screening and enrol eligible participants into the study. Included participants will then be allocated to a treatment group by two trained clinical investigators, who will administer the intervention and deliver the exercise education.

### Baseline assessment

i. Participant characteristics and anthropometrics

Anthropometric data, including weight and height, will be collected. Researchers will use structured questionnaires to obtain demographic and injury data, including presentation of symptoms (side of injury, dominant leg, pain location, duration of symptoms, perceived cause, previous diagnosis and treatment), level of physical activity (current and ultimate goal), presence of medical conditions (medical history and surgical history) and general health. The location of Achilles pain, pain quality and intensity will be measured via the digital application (Navigate Pain, Aglance Solutions, Aalborg, Denmark) [21].

For baseline testing, the painDETECT questionnaire will be implemented to screen neuropathic pain. This is a validated patient-reported measure that identifies the presence of neuropathic pain with high sensitivity, specificity, and positive predictive value [22]. The questionnaire is scored from zero to thirty-eight: a response between zero and twelve indicates that neuropathic pain is unlikely (<15%). A score of thirteen to eighteen is equivocal, and a score of nineteen or more indicates neuropathic pain is very likely (>90%).

ii. Plantar flexion contractile function

Plantarflexor maximal voluntary isometric contraction and rate of force development

Rate of force development (RFD) and maximal voluntary isometric contraction (MVIC) will be assessed using a custom-built ankle dynamometer. These measures intend to provide important information regarding neural and mechanical adaptations to load training [23]. Participants will be seated barefoot in the dynamometer with the ankle in plantargrade and knee joint flexed to 50° to optimise activation of soleus and gastrocnemius [24].

#### Plantarflexor force-matching the task

A force-matching task (FM) will be performed following the MVIC/RFD task in an identical position. Participants will be asked to maintain an ankle plantarflexor force equivalent to 10% of MVIC with visual feedback, a line indicating the target force, on a screen 1.5 m in front of them. Participants will be instructed to gradually reach the required force level and maintain this force for 15 s [25]. This force-matching task results in fluctuation around the target output, with smaller fluctuation indicating better muscle force control.

#### Test re-test reliability

A total of twelve healthy adult volunteers (seven males and five females, with a mean ± SD age of 37 ± 4.9 years, height 175 ± 10.8 cm, mass 65 ± 14.4.2 kg, BMI 21.1 ± 2.5) were recruited prior to the commencement of the study. Test-retest intra-rater reliability of diagnostic ultrasound imaging and the MVIC/RFD measures were determined across two sessions, 5 to 7 days apart. All measurements tested were on the right leg of the participants, and all demonstrated excellent reliability (ICCs >0.80 (95% confidence intervals 0.83 to 0.98) [26].

#### Plantarflexor six repetition maximum

Six repetition maximum (RM) of the seated and standing calf raise will be assessed bilaterally in random order, with 10 min rest in between. Repetition maximum is an accepted and reliable assessment of muscle strength [27]. Participants perform sets of six repetitions until a point of failure, establishing the maximum load for this number of repetitions; this commences at 80% bodyweight for seated exercises and 10% bodyweight for standing. Calf raise exercises will be standardised in terms of tempo, the height of the heel lift, the orientation of the tested foot, and upright trunk posture for standing exercises. For low-intensity groups, the baseline eighteen RM load will be estimated from six RM using the following formula: 6RM × 0.65 [28]. Week 1 exercise intensity will be adjusted down by 10% in both groups to minimise muscle soreness.

### Interventions

#### Exercise interventions

**Fig. 2:**
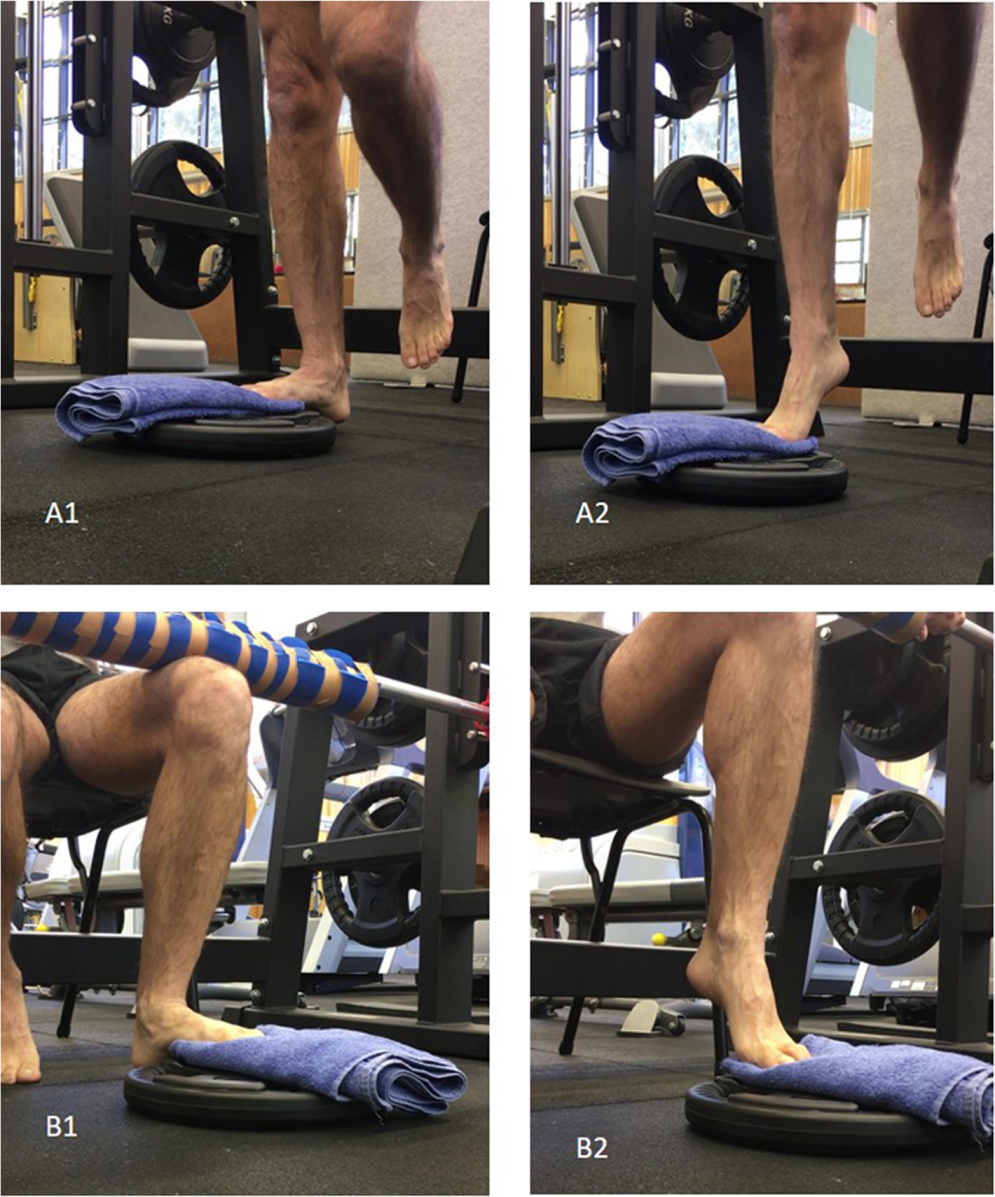
Calf raises in a Smith machine. A1) Starting position; A2) Ending position from standing; B1) Starting position; B2) Ending position from seated

Participants will be asked to perform two unilateral isotonic exercises that load the ankle plantarflexor complex at a local gymnasium. Exercises include a standing straight knee calf raise and a seated bent knee calf raise. Both exercises will be performed on a Smith machine for both legs, irrespective of unilateral or bilateral symptoms (Fig. 2).

The study groups will vary in terms of load intensity and time-under-tension (Table 1). Four sets of each exercise will be performed three times per week for 12 weeks, with standardised rest times. Participants will be taught to perform the calf exercise with appropriate fidelity, including a full available range of plantar flexion and dorsiflexion, performing the movement in the sagittal plane, smoothly and with appropriate tempo. Exercises will be externally paced with a metronome (an application downloaded to participants’ phones) to ensure that participants achieve the target exercise tempo.

**Table 1:** Calf exercise dosage for each group. RM repetition maximum; TUT time-under-tension; DF dorsiflexion; PF plantarflexion; min minutes; S seconds

	Group 1 High intensity with high time-under-tension	Group 2 High intensity with low time-under-tension	Group 3 Low intensity with high time-under-tension	Group 4 Low intensity with low time-under-tension
**Load intensity**	6 RM	6 RM	18 RM	18 RM
**Repetition**	6	6	18	18
**Sets**	4	4	4	4
**Frequency**	3×/week	3×/week	3×/week	3×/week
**Duration**	12 weeks	12 weeks	12 weeks	12 weeks
**Contraction time/rep**	3 s concentric 3 s eccentric	1 s concentric 1 s eccentric	3 s concentric 3 s eccentric	1 s concentric 1 s eccentric
**Rest in between reps**	No	No	No	No
**Rest in between sets**	2 min	2 min	2 min	2 min
**Contraction time per set**	36 s	12 s	108 s	36 s
**Total contraction time**	288 s	96 s	864 s	288 s
**Total loading time in session (with rests)**	16 min and 48 s	13 min and 36 s	19 min and 12 s	16 min and 48 s
**Volitional muscular failure**	Yes	Yes	Yes	Yes
**Range of motion**	0° to 15° DF 0° to 50° PF	0° to 15° DF 0° to 50° PF	0° to 15° DF 0° to 50° PF	0° to 15° DF 0° to 50° PF
**Time between sessions**	48 h	48 h	48 h	48 h
**Exercise form**	Seated and standing calf raising both performed on a Smith machine with barefoot	Seated and standing calf raising both performed on a Smith machine with barefoot	Seated and standing calf raising both performed on a Smith machine with barefoot	Seated and standing calf raising both performed on a Smith machine with barefoot

**Fig. 3:**
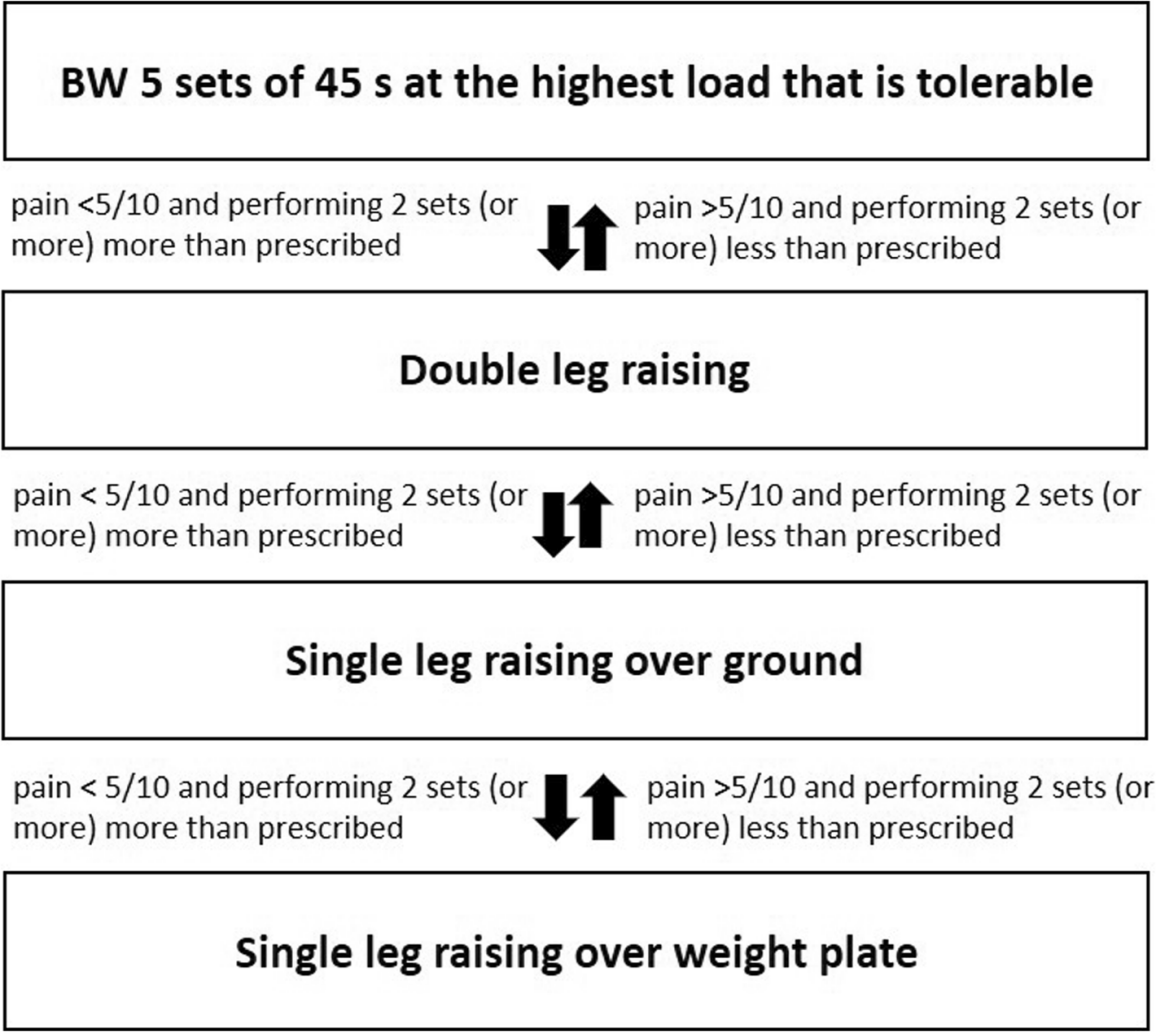
Load progression over 12 weeks of rehab period. BW: Body weight

Exercise load progression will be monitored weekly based on the participant’s pain and ability. The load will be based on the volitional muscular failure, where failure is defined as an inability to do another full repetition. The load will be influenced by the self-reported pain during the exercise, with less than five on an eleven-point numerical rating scale (where zero is no pain and ten is the worst pain imaginable) being defined as acceptable [29]. For participants who report their pain as a five or more during the exercise, the load will be reduced so that pain severity is within an acceptable range. If the pain is still not acceptable with minimal load, bodyweight in standing and unloaded in seated, the exercise will be performed on flat ground. Participants continuing to experience unacceptable pain will commence with isometric exercise. As the dorsiflexed position is likely to be pain provocative due to greater Achilles tendon load, these will be performed with the ankle in a unilateral mid-range plantarflexed position, or bilateral if too painful. Initially, participants will perform five sets of 45 s at the highest load tolerable, based on pain and ability to complete the exercise, until isotonic exercise can be performed with acceptable pain response as assessed on a weekly basis (Fig. 3).

Exercise load will be monitored at baseline and during the intervention period by tendon loading testing (i.e. single-leg submaximal hop or single-leg calf raise). The single-leg submaximal hop will be performed with standardised instructions: participants will be asked to hop continuously, with hands on their hips. The magnitude of Achilles pain will be measured on Numerical Rating Scale (NRS) where zero (the lower limit) represents no pain and ten (the upper limit) represents the worst pain level [30, 31]. It will then be compared between the affected, or most affected, and non-affected side.

### Physical activity modification

All participants will receive standardised advice regarding physical activity modification based on a pain-monitoring model [14]. This approach has previously been used in Achilles tendinopathy and produces comparable outcomes to complete rest from physical activity [14]. They also will receive individualised advice about progressing their sports activity. Participants will be advised to continue walking, running and participating in sports activity if Achilles tendon pain during these activities is not beyond a five out of ten on an eleven-point NRS (where zero is no pain and ten is the worst pain imaginable) [14]. It is acceptable for pain after activity to increase if it returns to pre-baseline levels on a tendon loading test, either a single leg submaximal hop or single leg calf raise, within approximately 24 h.

For some people who may have been performing Achilles loading activities daily, it may be difficult to determine the 24-h response to load. Therefore, we also will be using pain with load tests to adjust physical activity. If the worst load test pain is greater than five with either the hop or calf raise, we will advise a reduction in the most intense stretch-shorten cycle Achilles loading activity the person will be undertaking; typically, this is running or sport, but can be walking. The reduction will be by 20–50%, depending on symptoms and reassessed weekly by the load tests. Once the worst load test pain is again less than five, participants will be advised to increase gradually.

### Advice and education

Participants in each group will be given standardised information about Achilles tendinopathy, pathology, aetiology and management via a pamphlet. They will be informed that they will most likely experience muscle soreness within the first 2 to 4 weeks of the exercise program (delayed onset muscle soreness and/or fatigue) and given information about how to manage soreness. Participants will be requested to refrain from other forms of treatment during the study period. If they require medications for their Achilles tendon pain, they will be advised to consume up to 4 g/day pain-relieving medication, such as paracetamol or acetaminophen [32].

### Videoconference exercise monitoring

Exercise adherence and fidelity are critical to assess our aim of comparing exercise parameters between groups. To maximise this, we have developed a telerehabilitation protocol to monitor participants weekly. A practising physiotherapist (videoconference care provider) will be allocated to each participant at baseline and supervises exercise via a weekly videoconference session using Zoom (Zoom Video Communications, San Jose, California) for 12 weeks. The telerehabilitation protocol has been developed after extensive piloting to determine optimal camera position and angle, and the type of tripod suitable for the various Smith machines used in commercial gymnasiums. Bluetooth headphones will be supplied to participants and used during videoconferences, given the potential for disruptive noise in gymnasium environments.

During each weekly session, the videoconference care provider will collect adherence, health-care use, and adverse events data via questionnaire (Fig. 1). They will also observe and record the first set of each exercise—standing and seated on the right and left sides—without providing any feedback, assessing exercise fidelity. Real-time feedback will be provided during subsequent sets. Care providers will undergo 12 h of training using a variety of methods: one-to-one, online materials, and a group tendinopathy management master class covering up-to-date research and clinical perspective, as well as learning comprehensive rehabilitation strategies. They will also be provided with open access tendinopathy online training supervision materials, such as demo videos and screencasts.

### Primary feasibility outcomes: study processes

The following outcomes will be used to determine the feasibility of study processes for a randomised trial:

(i) Conversion, recruitment and retention rates. Conversation rate is the proportion of participants providing consent of those who met the selection criteria. Recruitment is the number of participants recruited per month. Retention is the proportion of recruited participants who complete the 12-week outcome assessment (primary endpoints).
(ii) Exercise adherence and fidelity. Exercise adherence and fidelity will be assessed from the information recorded by physiotherapists at weekly videoconference sessions. Adherence to exercise will be expressed as the percentage of prescribed exercise sessions completed or attempted per week. Exercise fidelity will be expressed as the percentage of the following key dose parameters: appropriate tempo per contraction, volume [repetitions and sets], intensity [failure +/−1 repetition].
(iii) Adverse events. The frequency (number of participants and number of cases), nature (e.g. rolled ankle, muscle tear or tendon pain worsening) and severity (mild, moderate or severe) will be recorded at each weekly videoconference session. People experiencing adverse events will be managed by the research team or triaged to an appropriate medical facility. The frequency of the use of paracetamol medication and other co-interventions to relieve Achilles pain will be also recorded.
(iv) Feasibility to collect economic outcomes. This will inform the decision to use an economic evaluation in a future definitive randomised trial. Health-related work productivity [33], as well as health-care use—namely current medications and treatment for Achilles—will be assessed at weekly intervals via a questionnaire during videoconference sessions.
(v) Participants’ and telerehabilitation care providers’ experiences with the interventions. At the end of the intervention period, two to three participants from each group will be invited to share their experience—the acceptability of interventions, and barriers and enablers to adherence—via qualitative interviews that will last for approximately thirty minutes. A focus group will be used to assess the views of videoconference care providers.

### Secondary clinical outcome measures

To obtain a wide variety of clinically relevant information regarding treatment effects, there are a number of secondary outcome measures: pain severity, health-related quality of life, perceived treatment response, physical activity, and psychological factors. These will be determined using patient self-reported questions at baseline, 6 and 12 weeks. Change in neuromuscular plantarflexor function will be assessed before and after 12 weeks of intervention. These outcomes were informed by core outcomes domains in tendinopathy process that two of the authors were involved with (PM, BV) [34].

(i) Pain and disability: The severity of pain and disability will be assessed using VISA-A, a disease-specific tool with acceptable construct validity and test-retest reliability [35]. This outcome includes pain, function and activity domains, the total score ranging from zero to one hundred (the latter indicating no symptoms and full function). One limitation of the VISA-A questionnaire is that it is designed for athletes. More specifically, the eighth question of the VISA-A, comprising up to 30% of the maximum possible score, is not applicable to non-athletes. As participants are athletes and non-athletes, we will use the original version of the VISA-A and a modified version of the VISA-A designed to be used for both athletes and non-athletes [36]. In the modified version, the word ‘sport’ has been replaced with ‘physical activity’, ensuring relevance to both sporting and non-sporting populations. We will subsequently assess the sensitivity to change of each version.

(ii) Activity pain: The worst pain imaginable level participants experience in the last week of the study will be assessed with an eleven-point rating scale, with zero being no pain and ten being the worst possible pain.

(iii) The patient impression of change: This will be assessed with the Patient Global Impression of Change (PGIC). A seven-point Likert scale is used to rate two questions: “How would you describe your Achilles tendon pain now, compared to before you began the treatment?”, and “How would you describe your ability to perform physical activities (such as walking, running, housework) now, compared to before you began the treatment?” For analysis purposes, the PGIC will be dichotomised so that “very much improved” and “improved” represents treatment effectiveness.

(iv) Participant satisfaction: Participant satisfaction or acceptability of symptom status will be measured using the Patient-Acceptable Symptom State instrument [37]. Participants will be asked: “Currently, are you satisfied with your condition?”, and “Would you recommend this treatment to another person who has Achilles pain?” Possible responses are yes or no.

(v) Health-related quality of life: This will be measured using the five-level EQ-5D version (EQ 5D 5 L), a validated and reliable tool among people with musculoskeletal pain [38]. The EQ 5D 5 L includes five domains—mobility, self-care, usual activities, pain/discomfort, and anxiety/depression—on a five-point Likert scale, and a rating of overall health state from zero (worst health state imaginable) to one hundred (best imaginable health state) using a VAS.

(vi) Level of physical activity: Participants’ physical activity will be measured using the seven-day Physical Activity Recall Questionnaire (PAR) [39], a valid and reliable measure of health-related physical activity behaviours in the previous seven days. The PAR has been used to assess physical activity behaviours in musculoskeletal and chronic pain populations [40].

(vii) Work productivity: This will be assessed with the Work Productivity and Activity Impairment Questionnaire (WPAI) that measures workdays lost. The WPAI is validated to measure impairments in work and activities [41]. The direct and indirect cost associated with delivery of the intervention may be an important measure for clinical decision making in Achilles tendinopathy rehabilitation.

(viii) Fear of movement: Kinesiophobia, or fear of movement/re-injury, will be measured with the Tampa Scale for Kinesiophobia (TSK) [42]. This questionnaire consists of seventeen statements rated on a four-point Likert scale (strongly disagree, disagree, agree, strongly agree). The total score ranges from 17 to 68, with higher scores indicating greater fear of movement.

(ix) Pain catastrophising: This will be measured using the Pain Catastrophising Scale (PCS) [43]. The PCS consists of thirteen items rated on a five-point Likert scale (not at all, to a slight degree, to a moderate degree, to a great degree, all the time) evaluating the degree to which participants experience catastrophic thoughts or feelings. The total score ranges from zero to 52, with higher scores indicating higher levels of pain catatrophisation.

(x) The exercise load (kg) for both seated and standing during calf raise exercises will be assessed at each weekly videoconference session. Set workload as a proportion of body mass (%) will be calculated through the 12 weeks of intervention (load (kg) × reps × time × sets = one exercise session).

### Sample size estimation

A formal sample size calculation was not performed, as this is a feasibility study and not subjected to hypothesis testing. Instead, we made a pragmatic decision that we would be able to achieve our secondary aim by recruiting forty-eight participants to be randomised into one of four factorial arms (*n* = 12 per trial arm as a rule of thumb recommended by Julious [44]).

### Data management and analysis

Both feasibility and clinical outcome data will be transferred to Excel spreadsheets, which will then be combined into one database. Quality checking for data entry will be performed by visually inspecting data and creating frequency tables for all items by two members of the research team. All data will be de-identified with analyses performed by an independent analyst. The randomisation code will not be broken until the final follow-up measurement has been performed (i.e. participant’s last visit), and data analysis has been completed. Both hard and soft copy data will be protected and accessible only to the research team. The principal investigator will have access to the final datasets. Results will be made available to participants on request and will be published in a peer-reviewed journal.

Means and standard deviations (or medians and IQRs, where data is not normally distributed), mean or median differences and their 95% confidence intervals of all outcome measures will be calculated at each follow-up time. Point estimates of effect will be calculated as the difference between group means expressed as a proportion of the pooled standard deviation. The effect size criteria will be interpreted as per Hopkins [45], with very large being ≥1.2, moderate being ≥0.6, and small being ≥0.2. Standard tests to assess continuous data for normal distribution will be used and transformation will be carried out if required for further analysis.

The MVIC force data (Nm) will be analysed directly from PowerLab (AD Instruments Corp, Dunedin, NZ) for each recorded trial. RFD data (N·s-1) and force match data will be exported to Excel (Microsoft Corporation, Redmond, WA). The RFD will be analysed using a custom-written software program (rehabtools.org, Sunshine Coast, Australia). For the FM task, a sample of force (from five to fifteen seconds during each trial) will be obtained to calculate the mean and coefficient of variation (CV) of force (SD/mean force % 100).

The trial will be deemed a success based on the following:

(i) Randomising 20% or more of eligible patients that fit the selection criteria.
(ii) Achieving 66% or more of the number of sessions completed in each group.
(iii) Achieving 66% or more of intervention fidelity (i.e. prescribed dose parameters including tempo, volume, and intensity in each group).
(iv) Report of ≤5% of serious adverse events.

## Discussion

Although calf loading exercise is widely recognised as an important intervention for Achilles tendinopathy, there is very little evidence regarding the optimal exercise dose to improve clinical outcomes. Exercise is a first-line recommended treatment for Achilles tendinopathy [20], but there is confusion among clinicians about the parameters that should be prescribed [46]. This most likely stems from very heterogeneous literature. Among trials investigating exercise interventions, there are heterogeneous participant characteristics (including gender and activity levels), exercise parameters, outcomes and study quality, especially when it comes to reporting adherence and load progression. Therefore, clinicians do not currently have clear guidance regarding particular exercise parameters or approaches that may be suitable for people with Achilles tendinopathy.

The proposed study and follow-up powered randomised trial will be a first step towards determining exercise dose parameters that may optimise outcomes for Achilles tendinopathy. It is well established that the tendon is a mechanoresponsive tissue and tendon strain applied for a sustained period provides a stimulus for adaptation—for example, increased stiffness. The sustained strain is mediated by load intensity and time-under-tension. Therefore, we have chosen to focus on these parameters, as they are important for tendon adaptation and may confer benefits for people with Achilles tendinopathy. This work has the potential to lead to more effective exercise loading interventions for Achilles tendinopathy.

A factorial design has been chosen for two main reasons. First, it is not possible to compare load in a two-arm design without one other characteristic varying. If one group performed four sets of six repetitions and the other performed four sets of eighteen, then the time-under-tension is greater in the latter group. This can be controlled by asking the second group to also perform four sets of six at an eighteen RM intensity; however, the varying factor then becomes whether people are achieving fatigue or not. The factorial design allows researchers to have two groups matched for time-under tension. Second, the factorial designs allow comparison of outcomes of a different combination of time-under-tension and load intensity. We recognise that in this feasibility pilot, we are not powered to investigate effects between the different groups, but it is important to justify our choice of factorial design.

The outcome measures we will use in this trial have been informed by the recent development of a core outcome set for tendinopathy that two of the authors were involved with. We have included outcomes reflecting all of the core outcome domains, including a validated and disease-specific pain and disability outcome (VISA-A). Two versions of the VISA-A will be included: the original, and an amended version that may be more suitable to non-athlete participants.

## Trial Status

The trial commenced in July 2018; recruitment for the study is ongoing. The trial is funded to run up to 2020.

## Data Availability

Not applicable.

## Abbreviations

LOADIT: LOAD Intensity and Time under tension trial
VISA-A: Victorian Institute of Sports Assessment–Achilles questionnaire
MVIC: Maximal Voluntary Isometric Contraction
RFD: Rate of Force Development
FM: Force-matching task
ICC: Intraclass Correlation Coefficient
NRS: Numerical Rating Scale
BMI: Body mass index
PGIC: Patient Global Impression of Change
WPAI: Work Productivity and Activity Impairment Questionnaire
